# Molecular tools confirm natural *Leishmania* (*Viannia*) *guyanensis/L.* (*V.*) *shawi* hybrids causing cutaneous leishmaniasis in the Amazon region of Brazil

**DOI:** 10.1590/1678-4685-GMB-2020-0123

**Published:** 2021-04-30

**Authors:** Ana Carolina S. Lima, Claudia Maria C. Gomes, Thaise Y. Tomokane, Marliane Batista Campos, Ricardo A. Zampieri, Carolina L. Jorge, Marcia D. Laurenti, Fernando T. Silveira, Carlos Eduardo P. Corbett, Lucile Maria Floeter-Winter

**Affiliations:** 1 Universidade de São Paulo, Faculdade de Medicina, Departamento de Patologia, São Paulo, SP, Brazil.; 2 Universidade de São Paulo, Instituto de Biociências, Departamento de Fisiologia, São Paulo, SP, Brazil.; 3 Ministério da Saúde, Secretaria de Vigilância em Saúde, Instituto Evandro Chagas, Belém, PA, Brazil.

**Keywords:** Cutaneous Leishmaniaisis, hybrid parasite, L. (V.) guyanensis, L. (V.) shawi, Brazil

## Abstract

Seven isolates from patients with American cutaneous leishmaniasis in the Amazon region of Brazil were phenotypically suggestive of *Leishmania (Viannia) guyanensis*/*L*. *(V.) shawi* hybrids. In this work, two molecular targets were employed to check the hybrid identity of the putative hybrids. Heat shock protein 70 (*hsp70*) gene sequences were analyzed by three different polymerase chain reaction (PCR) approaches, and two different patterns of inherited *hsp70* alleles were found. Three isolates presented heterozygous *L*. (*V.*) *guyanensis*/*L*. (*V*.) *shawi* patterns, and four presented homozygous *hsp*70 patterns involving only *L*. (*V*.) *shawi* alleles. The amplicon sequences confirmed the RFLP patterns. The high-resolution melting method detected variant heterozygous and homozygous profiles*.* Single-nucleotide polymorphism genotyping/cleaved amplified polymorphic site analysis suggested a higher contribution from *L*. (*V.*) *guyanensis* in *hsp70* heterozygous hybrids. Additionally, PCR-RFLP analysis targeting the enzyme mannose phosphate isomerase (*mpi*) gene indicated heterozygous and homozygous cleavage patterns for *L*. (*V*.) *shawi* and *L*. (*V*.) *guyanensis*, corroborating the *hsp70* findings. In this communication, we present molecular findings based on partial informative regions of the coding sequences of *hsp70* and *mpi* as markers confirming that some of the parasite strains from the Brazilian Amazon region are indeed hybrids between *L. (V.) guyanensis* and *L. (V.) shawi*.

## Introduction

The genus *Leishmania* presents highly diverse nonsexual mechanisms for the generation of diversity*.* These mechanisms include the occurrence of tandem repeat genes, gene amplification, gene duplication, mini-chromosome generation and mosaic aneuploidy (Delgado et al., 1997; Victoir and Dujardin, 2002; Dujardin *et al.,* 2007; Sterkers et al., 2011). This high genome plasticity is achieved through both sexual-like and nonsexual characteristics, providing great complexity that is reflected in the wide geographical distribution of *Leishmania* spp., the diversity of its hosts and the complexity of illnesses associated with the parasites.

New tools for the study of the reproduction mechanisms of the *Leishmania* genus have expanded the knowledge of these mechanisms, showing that parasite population structure is predominantly clonal, but rare sexual events can occur (Tibayrenc and Ayala, 1991; Tibayrenc *et al.,* 1991). Nevertheless, genetic exchange between *Leishmania* parasites as well as other related trypanosomatids has been experimentally demonstrated, and complex analyses have been performed in hybrid strains (Gaunt et al., 2003; Akopyants et al., 2009; Rogers et al., 2014; Inbar et al., 2019). 

Reports of hybrids occurring in natural environments or involved in cases of leishmaniasis are recurring worldwide. In some cases, these hybrids are associated with severe forms of the disease, including the mucosal form found in Peru (Dujardin et al., 1995). The occurrence of hybrids has been directly correlated with areas of sympatric species occurrence, which enables the interaction of various genotypes in coinfected vectors (Inbar et al., 2013; Kato et al., 2019). Studies have shown that significant inbreeding of parasites from strains that are genetically related or even identical can occur in the vector host (Rougeron *et al.,* 2009). The low frequency of coinfection in the invertebrate host represents the major barrier to mating between unrelated strains (Bastien et al., 1992; Inbar *et al.,* 2013). Recent studies have shown that intraspecies hybrids are prone to hybridization events, while interspecies hybrids seem to be sterile (Inbar, *et al.,* 2019).

In the lower Amazon region of Pará State, Brazil, reports indicate the circulation of at least five different species of *Leishmania,* involved with American cutaneous leishmaniasis (ACL) cases, in addition to two different subpopulations of *L. (V.) shawi,* classified as *L. (V.) shawi shawi* and *L. (V.) shawi santarensis* (Jennings et al., 2014). The authors also isolated seven atypical strains from human cases of ACL from Santarém, a city located in the lower Amazon region. By means of multilocus enzyme electrophoresis (MLEE) and monoclonal antibody analysis, these atypical strains presented phenotypic profiles suggestive of hybridization between the species *L*. *(V.) guyanensis* and *L. (V.) shawi* (Table S1). 

The verification of the *L*. *(V.) guyanensis/ L*. *(V.) shawi* hybrid parasites involved in ACL cases in the Santarém region updates the eco-epidemiological scenario in this important endemic area of Brazil. Therefore, the aim of the present study was to search for evidence of genetic recombination in these strains using *hsp70* sequences as targets for polymerase chain reaction and restriction fragment length polymorphisms (PCR-RFLP), single-nucleotide polymorphism genotyping/cleaved amplified polymorphic site (SNP-CAPS), high-resolution melting PCR (HRM) and PCR product sequencing analyses. An additional PCR-RFLP analysis of *mpi* sequences for comparison with *hsp70* findings was performed. Together, our findings confirmed by molecular methods that five of seven isolates presented genetic traces of hybrid strains between *L*. (*V*.) *shawi* and *L*. (*V*.) *guyanensis.*

## Material and Methods

### Parasites

The *Leishmania* strains were obtained from the Evandro Chagas Institute (Surveillance Secretary of Health, Ministry of Health), Pará State, Brazil and are listed in Table 1. These strains included 21 reference strains of the main species present in the lower Amazon region, including four different reference strains of *L*. *(V.) guyanensis*, two of *L*. *(V.) shawi shawi*, two of *L*. *(V.) shawi santarensis* and the seven putative *L*. *guyanensis* /*L*. *shawi* hybrids that were isolated from the skin lesions of patients with localized cutaneous leishmaniasis.

**Table 1 -:** Leishmania strains used in this study.

***Leishmania* sp.**	Reference strain	Geographical origin
*Leishmania* (*Leishmania*) *amazonenses*	MHOM/BR/71973/M2269	Cafezal - Pará State
*Leishmania* (*Leishmania*) *chagasi*	MCER/BR/1981/M6445	Salvaterra - Pará State
*Leishmania* (*Leishmania*) *mexicana*	MNYC/BZ/62/M379	Belize
*Leishmania* (*Viannia*) *braziliensis*	MHOM/BR/1975/M2903	Parauapebas - Pará State
*Leishmania* (*Viannia*) *guyanensis*	MHOM/BR/1775/M4147	Monte Dourado - Pará State
*Leishmania* (*Viannia*) *guyanensis*	MHOM/BR/1990/M13245	Óbidos - Pará State
*Leishmania* (*Viannia*) *guyanensis*	MHOM/BR/1997/M16174	Óbidos - Pará State
*Leishmania* (*Viannia*) *guyanensis*	MHOM/BR/2001/M19869	Óbidos - Pará State
*Leishmania* (*Viannia*) *lainsoni*	MHOM/BR/1981/M6426	Benevides - Pará State
*Leishmania* (*Viannia*) *naiffi*	MHOM/BR/1979/M5533	Jari- Pará State
*Leishmania* (*Viannia*) *shawi shawi*	MCEB/BR/1984/M8408	Serra dos Carajás - Pará State
*Leishmania* (*Viannia*) *shawi shawi*	MHOM/BR/2001/M19664	Alenquer - Pará State
*Leishmania* (*Viannia*) *shawi santarensis*	MHOM/BR/1996/M15982	Santarém - Pará State
*Leishmania* (*Viannia*) *shawi santarensis*	MHOM/BR/1996/M15985	Santarém - Pará State
*Leishmania* (*Viannia*) spp.	Characterized strain^1^	Geographical origin
*L*.(*Viannia*) sp.	MHOM/BR/1996/M15983	Santarém - Pará State
*L*.(*Viannia*) sp.	MHOM/BR/1996/M15984	Santarém - Pará State
*L*.(*Viannia*) sp.	MHOM/BR/1996/M15987	Santarém - Pará State
*L*.(*Viannia*) sp.	MHOM/BR/1996/M15988	Santarém - Pará State
*L*.(*Viannia*) sp.	MHOM/BR/1996/M19672	Santarém - Pará State
*L*.(*Viannia*) sp.	MHOM/BR/1996/M19676	Santarém - Pará State
*L*.(*Viannia*) sp.	MHOM/BR/1996/M19697	Santarém - Pará State

1: *L.* (*V*.) *guyanensis*/*L. (V.) shawi* hybrids

### Cloning of the parasites

Two different methodologies were used for *Leishmania* spp. cloning.

The cloning protocol involved plating the parasites in solid growth media according to Muniaraj et al. (2010) with some modifications. We chose to prepare the culture plates using 0.5 volumes (V) of 2x Noble agar medium (Sigma; St. Louis, MO, USA) and 0.5 V of 2x Schneider medium (Sigma; St. Louis, MO, USA), with a final fetal bovine serum (FBS) concentration of 10%. Then, 10^3^ cells were seeded per plate, followed by incubated at 25 °C for 30 days.

The second cloning method was performed according to Handman et al. (1983) with modifications that are briefly described here. The parasites were cultivated in Schneider´s insect medium with 10% FBS, and stationary-phase cultures were cloned via limiting dilution assays at concentrations from 6.2 x 10^4^ to 0.03 parasites/mL. Cultures were expanded from wells containing approximately one parasite per mL.

### DNA extraction

DNA was obtained from each culture by phenol-chloroform extraction followed by precipitation with sodium acetate and ethanol (Uliana et al., 1991), then resuspended in Tris-EDTA buffer (10 mM Tris-HCl, 1 mM EDTA, pH 7.4).

### DNA and PCR product quantification

The purified PCR products and DNA obtained from the parasite cultures were quantified using a BioSpectrometer (Eppendorf) according to the manufacturer’s instructions.

### *hsp70* PCR-RFLP analysis

DNA from five clones from each isolate strain and the reference strains was used in the PCR assays. Each PCR assay was performed in a final volume of 50 µL containing 50 ng of DNA, each primer at 0.2 µM and 25 µL of Top Taq Master Mix (Qiagen, Germantown, USA). The primers used for *hsp70* were *hs*p*70c* forward, 5’-GGACGAGATCGAGCGCATGGT-3’, and *hsp70c* reverse, 5’-TCCTTCGACGCCTCCTGGTTG-3’, which amplify a 234 base pair (bp) fragment (Graça et al., 2012). The PCR assays were conducted with the following amplification program: 94 °C for 5 min, by 40 cycles of 94 °C for 1 min, 64 °C for 1 min and 72 °C for 30 s, with a final extension at 72 °C for 10 min. The reaction product was analyzed by electrophoresis on a 2% agarose gel in TAE buffer (40 mM Tris-acetate and 2 mM EDTA). DNA fragments were stained with Gel Red (Biotium, Freemont, CA, USA) and were visualized in a transilluminator Gel Logic 212 Pro^®^ system (Carestream Molecular Imaging; Woodbridge, CT, U.S.A) at 260 nm. Images were captured with a Pro imaging GL212 camera using an orange filter.

The *hsp70* PCR-RFLP analysis for discrimination between *L. (V.) guyanensis* and *L. (V.) shawi* was performed as described by Graça et al. (2012). The PCR products were purified using a GeneJet PCR purification Kit (Thermo Fisher Scientific, Waltham, MA, USA), and 300 ng of purified PCR product was digested with 10 units (U) of *Hae*III (Thermo Scientific-Walthman, MA, USA) using the conditions recommended by the manufacturer. Incubation was performed at 37 °C for 12 hours. The digested products were subjected to 3% agarose gel electrophoresis and stained with GelRed. The visualization of fragments was performed as described above.

### *mpi* PCR-RFLP analysis

The *mpi* PCR-RFLP analysis for discrimination between *L. (V.) guyanensis* and *L. (V.) shawi* was performed as described by Boité et al. (2012). DNA from five clones from each isolate strain and the reference strains were used for PCR. Each amplification reaction was performed in a final volume of 50 µL containing 50 ng of DNA, each primer at 2 µM and 25 µL of Top Taq Master Mix (Qiagen, Germantown, USA). The primers used were *mpi* forward, 5’- GGCAAGATGTATGCGGAGTT-3’, and *mpi* reverse 5’-TCCTTCGACGCCTCCTGGTTG-3’, which amplify a 681 bp fragment. The PCR assays were performed with the following amplification program: 94 °C for 5 min, followed by 35 cycles of 94 °C for 1 min, 58 °C for 1 min and 72 °C for 30 s, with by a final extension at 72 °C for 10 min. The reaction product was analyzed by electrophoresis on a 2% agarose gel in TAE buffer (40 mM Tris-acetate and 2 mM EDTA). DNA fragments were stained with Gel-Red and were visualized in a transilluminator Gel Logic 212 Pro® system at 260 nm. Images were captured with a Pro imaging GL212 camera using an orange filter.

The PCR products were purified using a GeneJet PCR purification Kit (Thermo Fisher Scientific, Waltham, MA, USA), and 300 ng of the purified PCR product was digested with 1 U of *Cla* I (New England BioLabs- Ipswich, MA, EUA) under the conditions recommended by the manufacturer. Incubation was performed at 37 °C for 2 h. The enzyme was heat inactivated at 65 °C for 15 min. The digested products were subjected to 2% agarose gel electrophoresis and stained with GelRed. The visualization of fragments was performed as described above.

### Cloning and sequencing of *hsp70* PCR products

The *hsp70*c PCR products were ligated into the PGEM T Easy vector (PROMEGA-Madison, Wisconsin, EUA) and cloned into *E. coli* bacteria (SURE). The *hsp70* amplicons were sequenced by Sanger sequencing (Big Dye Terminator V3.1 cycle sequencing kits; Applied Biosystems). PCR product sequencing was performed for five putative *Leishmania* hybrid isolates (isolates 2 to 6) as well as two strains of *L. (V.) guyanensis* and *L. (V.) shawi.* Three independent amplification reactions were performed for each isolate. Six plasmids containing PCR product clones were chosen from each PCR assay, from which four PCR product clones were sequenced.

### SNP-CAPS analysis of putative hybrids and progeny lines

The proportion of each *hsp70* allele in each isolate was determined by comparing the relative intensity of the PCR products between putative hybrids and progeny lines after SNP-CAPS analysis (Akopyants et al., 2009). The primers and PCR conditions were the same as those indicated above. A total of 300 ng of the purified *hsp70* amplicon from the putative hybrid or parental reference strain was cleaved using 10 U of *Hae*III at 37 °C for 12 h. The intensity of the bands was compared to three amplicon mixtures of 300 ng of the purified *hsp70* PCR products from the DNA of the parental strains *L. (V.) guyanensis* and *L. (V.) shawi* at proportions of 2:1, 1:1 and 1:2, respectively. An analysis of the relative intensity of bands was performed using Carestream Molecular Imaging Software.

### HRM analyses

The hybrids isolates were evaluated via HRM analyses to observe possible discriminatory patterns capable of indicating allelic variations. Real-time PCR assays were performed as described by Zampieri et al. (2016) in PikoReal96 thermocycler (Thermo Fisher Scientific, Waltham, MA, USA) using MeltDoctor HRM Master Mix (Life Technologies; Carlsbad, California, USA) with 50 ng of genomic DNA as the template and the *hsp70F2* (5’-GGAGAACTACGCGTACTCGATGAAG-3’) and *hsp70c* reverse primers at 200 nM, as described above. The cycling conditions were 94 °C for 5 min, followed by 40 cycles of 94 °C for 30 s and annealing/extension at 60 °C for 30 s, with fluorescent signal acquisition at the end of each extension step, followed by the dissociation curve for HRM analysis. The amplicon dissociation analyses were performed via the acquisition of fluorescence signals at 0.2 °C intervals with holding for 10 s between 60 °C and 95 °C. Genomic DNA samples from the reference strains *L. (L.) infantum chagasi* (MCER/BR/1981/M6445), *L. (L.) amazonensis* (MHOM/BR/1973/M2269), *L. (L.) mexicana* (MNYC/BZ/62/M379), *L. (L.) lainsoni* (MHOM/BR/81/M6426), *L. (V.) braziliensis* (MHOM/BR/1975/M2903), *L. (V.) guyanensis* (MHOM/BR/1975/M4147), *L. (V.) naiffi* (MDAS/BR/1979/M5533) and *L. (V.) shawi* (MCEB/BR/84/M8408) were used as standards, and the HRM profiles generated for these species were the benchmark of the analysis.

## Results

### Homozygous or heterozygous RFLP profiles were detected in hybrid strains

**Figure 1 -:**
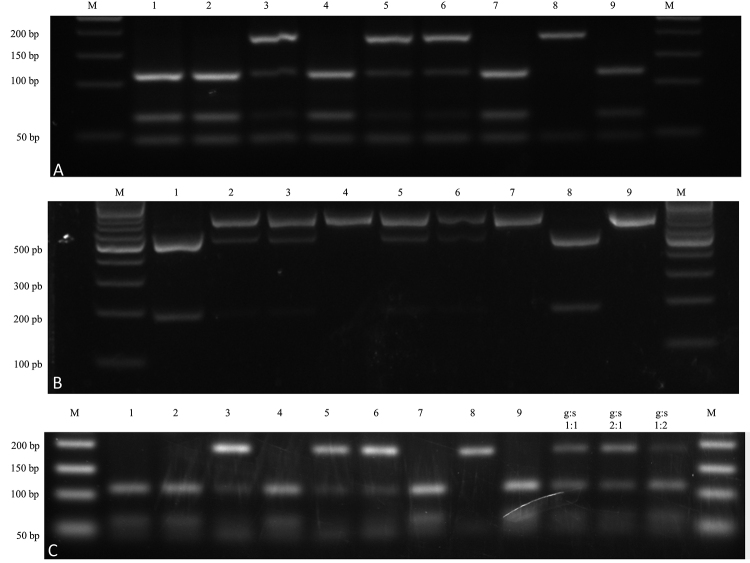
Electrophoresis gel fractionated products on agarose stained with GelRed showing homozygous and heterozygous PCR-RFLP patters. **A:** *hsp*70 PCR-RFLP (*Hae*III). M: 50 bp molecular weight marker (Fermentas SM 0373). Lanes 1 to 7: isolates 1 to 7, respectively. Lane 8: *L. (V.) guyanensis* (MHOM/BR/1975/M4147). Lane 9: *L. (V.) shaw shawi* (MCEB/BR/1984/M8408). **B:** *mpi* PCR-RFLP (*Cla* I). M: 100 bp molecular weight marker (Fermentas SM 0331). Lanes 1 to 7: isolates 1 to 7, respectively. Lane 8: *L. (V.) guyanensis* (MHOM/BR/1975/M4147). Lane 9: *L. (V.) shaw shawi* (MCEB/BR/1984/M8408). **C:** SNP-CAPS *hsp70* PCR-RFLP (*Hae*III). All lanes contain 300 ng of purified PCR products digested by *Hae*III. Lanes 1 to 7: hybrids *Leishmania* (*Viannia*) spp. isolates 1 to 7, respectively. Lane 8: *L. (V.) guyanensis*; Lane 9: *L. (V.) shawi shawi;* Lane 10: *L. (V.) guyanensis*: *L. (V.) shawi shawi* (1:1); Lane 11: *L. (V.) guyanensis*: *L. (V.) shawi shawi* (2:1); Lane 12: *L. (V.) guyanensis*: *L. (V.) shawi shawi* (1:2); and M: 50 bp molecular weight marker.

The analysis of the results of the *Hae*III digestion of the *hsp70* PCR products and the *Cla* I digestion of the *mpi* PCR products was performed for seven isolates (Figure 1A and Figure 1B), as well as for five clones obtained from each isolate (Figure S1). It was shown, that the hybrid phenotype of the isolates was not due to mixed infection, since all the clones from each isolate presented the same cleavage profiles in RFLP assays (Figure S1).

*Hae*III cleavage of the PCR products generated three fragments of 47, 67 and 120 base pairs for *L. (V). shawi* and two fragments of 47 and 187 base pairs for *L. (V.) guyanensis* as shown by the *hsp70-RFLP* analysis of these putative parental strains. Four different strains of these species from the same geographic area were included to assure more consistent results (Figure S1). The *hsp70* PCR products obtained from the seven isolates generated two RFLP profiles, one that was characteristic of heterozygous alleles, while the other was characteristic of homozygous profile of the *L. (V). shawi* alleles (Figure 1A).

Three isolates (isolates 3, 5 and 6) showed a heterozygous profile resembling the pattern for *L. (V.) shawi* and *L. (V.) guyanensis* with fragments characteristic of both species (Figure 1A). The isolates 1, 2, 4 and 7 presented the *hsp70* pattern that was compatible with the homozygous profile of the *L. (V). shawi* alleles (Figure 1A). To verify these polymorphic profiles in more detail, the *hsp70c* PCR products were cloned and sequenced. The restriction sites of the *Hae*III enzyme in the sequences corresponded to the cleavage pattern obtained in the RFLP assay (Figure S2). Five isolates were chosen for sequencing: three from the heterozygous *hsp70* profile and two from the homozygous *hsp70* isolates. The *hsp70* PCR nucleotide sequences were deposited in GenBank with the accession numbers MT337389 to MT337400 (Table S2). The nucleotide sequences of the *hsp70* PCR products obtained from the heterozygous *hsp70* strains presented profiles corresponding to *L. (V.) shawi* and *L. (V.) guyanensis*, as could be observed from the cloned amplicons of the same sample (Figure S2). The presence of both alleles confirmed the PCR-RFLP pattern presented by the heterozygous isolates. The homozygous cloned PCR nucleotide sequences presented only *L. (V) shawi* alleles.

*Cla I* cleavage did not cut the *mpi* PCR product of *L*. (*V*.) *shawi* and generated two fragments for *L. (V.) guyanensis* of 184 and 497 base pairs. Two of seven isolates (isolates 4 and 7) presented homozygous profiles compatible with *L. (V.) shawi.* One isolate (isolate 1) presented a homozygous profile compatible with *L. (V.) guyanensis*. Four of seven isolates (isolates 2, 3, 5 and 6) presented a heterozygous hybrid pattern of *mpi* alleles (Figure 1B). Altogether, the *hsp70* and *mpi* PCR-RFLP data reinforced the phenotypic findings favoring the hybrid identity for five of seven (5/7) of these isolates (Figure 1, Figure S1 and Table 2). The seven isolates were divided into four groups when the molecular targets were analyzed together.

**Table 2 -:** Isolates profile according to *hsp70* and *mpi* PCR-RFLP.

Isolate	***L*. (*Viannia*) spp.**	*hsp70*	*mpi*
	*L*. (*V*.) *guyanensis*	*L*.(*V*.) *g*.	*L.*(*V*.) *g*.
	*L*. (*V*.) *shawi*	*L*.(*V*.) *s.*	*L*.(V.) *s*.
1	M15983	*L*.(*V*.) *s.*	*L*.(*V*.) *g.*
2	M15984	*L*.(*V*.) *s.*	hybrid
3	M15987	hybrid	hybrid
4	M15988	*L*.(*V*.) *s.*	*L*.(*V*.) *s.*
5	M19672	hybrid	hybrid
6	M19676	hybrid	hybrid
7	M19697	*L*.(*V*.) *s.*	*L*.(*V*.) *s.*

*hsp70*= heat shock protein 70; *mpi* = mannose phosphate isomerase; *L. (V.)g.: L.* (*V.*) *guyanensis; L. (V.) s: L.* (*V.*) *shawi;* hybrid: heterozygous cleavage pattern.

### *hsp70* SNP-CAPS analysis of the putative hybrids

To perform a qualitative analysis of the proportion of parental loci in the *hsp70* inheritance profile of the *hsp70* hybrid strains, SNP-CAPS assays were performed, allowing a comparative analysis of the intensity of the generated fragments with the parental and uncloned hybrid strain profiles.

The putative *hsp70* homozygous hybrids presented bands with intensity similar to that in *L. (V.) shawi*, suggesting an equivalent number of *hsp70* copies. The putative *hsp70* heterozygous showed fragments corresponding to *L. (V.) guyanensis* with higher intensities (Figure 1C). The analysis of the relative intensity of the bands was performed using Carestream Molecular Imaging Software in relation to the heterozygotes, showing that the *L. (V.) guyanensis* bands were approximately six times brighter those of the heterozygous isolates (Figure S3).These results suggest that *L. (V.) guyanensis* may have made a greater contribution to the inheritance of these alleles.

### HRM analysis of *hsp70* real-time PCR products corroborated the RFLP results

**Figure 2 -:**
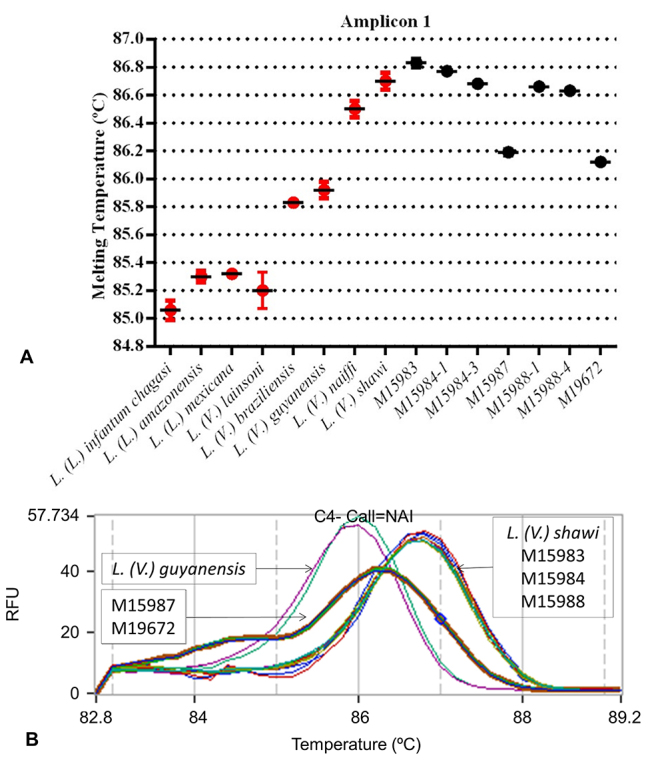
HRM using *hsp70* as target**. A-** Representative dispersion graph shows the average and standard deviation of the T_m_ values. **B**-Normalized melting curves for *L. (V.) guyanensis, L. (V.) shawi* and hybrid isolates 1, 2, 3, 4 and 5.

The evaluation of polymorphic nucleotide sequences by HRM analysis revealed differences between the parental and hybrid haplotypes in five putative hybrids isolates (1, 2, 3, 4 and 5). One of the main parameters used in the analysis was the melting temperature (Tm). Discrete variations in the nucleotide composition of DNA fragments are reflected in Tm variations. The Tm values for the *L. (V.) shawi* and *L. (V.) guyanensis hsp70* PCR products were 86.80 °C and 86.14 °C, respectively (Figure 2A and Figure 2B). The hybrid parasites with a homozygous *hsp70* profile showed a dissociation temperature and melting curve corresponding to *L. (V.) shawi* (Figure 2A and Figure 2B). The *hsp70* heterozygous hybrids showed a unique dissociation temperature and a melting curve distinct from those of all other examined species.

## Discussion

The strains characterized in the present study were isolated from patients inhabiting the lower Amazon region in Pará State, an important endemic area of leishmaniasis in Brazil. The identification of seven different *Leishmania* strains involved in ACL presenting high intraspecific diversity in this geographic area is relevant to epidemiological studies (Jennings et al., 2014; de Souza et al., 2016).

Santarém is also an important endemic area of visceral leishmaniasis (Braga et al., 1986). The considerable diversity of vectors and reservoirs in the Amazon rain forest, one of the most complex, diverse biomes on the planet, is also notable (Christensen et al., 1982; Grimaldi et al., 1991; Silveira et al., 1991; Lainson et al., 1994).

Due to the species diversity observed in Santarém, each isolate was initially cloned to eliminate the possibility of a mixed infection.

The patterns of 234 bp fragments from *hsp70* PCR-RFLP analysis yielded two profiles. One was similar to that of the parental species *L*. (*V*.) *shawi,* which was considered a homozygous pattern. The other pattern showed bands characteristic of both *L. (V.) shawi* and *L. (V.) guyanensis* and was considered a heterozygous profile, which is commonly used parameter to confirm hybridization events between species (Gaunt et al., 2003; Inbar et al., 2013). According to studies that have investigated *Leishmania* mating, the inheritance profile of the parasite consists of the same number of chromosomes derived from each parent during a sexual reproduction event, generating heterozygous hybrids (Akopyants et al., 2009; Rogers et al., 2014). The PCR-RFLP analysis performed for *mpi* alleles showed heterozygous or homozygous profiles for *L. (V.) shawi* or *L. (V.) guyanensis*. These findings reinforced the previous phenotypic findings and, together with the *hsp70* results, confirmed the hybrid nature of these isolates.

The isolates 4 and 7 presented *hsp70* and *mpi* alleles compatible with *L. (V.) shawi* (Table S1). However, isoenzyme analyses showed that these strains harbor at least two isoenzymes compatible with *L. (V.) guyanensis*, which strongly suggest the hypothesis of their hybrid nature, as demonstrated in Table S1. In addition, the three band profile observed for the dimeric 6PGD isoenzyme via MLEE analysis was associated with a heterozygous hybrid profile (Jennings et al., 2014). Similar findings were reported for *L. (V.) braziliensis /L. (V.) guyanensis* hybrids from Venezuela (Dujardin et al., 1995; Delgado et al., 1997). Rogers et al. (2014) proposed that after an initial mating event, the hybrid cells participate in inbreeding events exclusively among themselves. This hypothesis could justify the homozygous profile found for some isolates because a high endogamy rate can lead to loss of heterozygosity (Inbar et al., 2013; Gelanew et al., 2014; Rogers *et al.,* 2014). Another important aspect that must be considered in relation to the homozygous profile is mosaic aneuploidy, a widespread characteristic of the genus *Leishmania* (Rogers *et al.,* 2011; Sterkers et al., 2012)*.* This phenomenon is probably caused by a chromosomal replication defect, followed by asymmetric segregation that occurs rapidly and that leads to the loss of heterozygosity (Sterkers *et al.,* 2011; Inbar *et al.,* 2013). This factor cannot be ignored because hybrid strains are established by clonal propagation and are therefore subject to the events associated with such mechanisms.

Four isolates presented a heterozygous hybrid profile for at least one analyzed molecular target and one isolate presented *hsp70* heritage from *L*. (*V*.) *shawi* and *mpi* from *L*. (*V*.) *guyanensis*. Altogether, these molecular analyses confirmed that at least five isolates are true *L*. (*V*.) *shawi* and *L*. (*V*.) *guyanensis* hybrids. 

Considering the variable isoenzyme plus *hsp70* and *mpi* RFLP profiles, the seven isolates were divided into six different groups (Table S1). Such diversity among hybrid isolates is supported by the hybridization and recombination model proposed by Rogers et al. (2014). This could suggest that hybridization events may be advantageous for a genus, offsetting the predominant mode of clonal reproduction to some extent. It is also notable interesting to note that different hybrid strains were found in patients with ACL, reflecting a well-established cycle in the environment. More information about the niche occupied by different hybrids in the environment would help to elucidate the interactions among the various genotypes and their establishment in the environment. A previous study suggested that *Nyssomyia whitmani* can act as permissive vector, in which mating events between *L. (V.) shawi* and *L. (V.) guyanensis* could occur (de Souza et al., 2016).

The SNP-CAPS analysis showed a similar intensity between the putative homozygous *hsp*70 hybrids and the *L. (V.) shawi hsp70* fragment, suggesting that they have the same number of copies of this gene. In relation to the heterozygous *hsp70* profile, the SNP-CAPS assay suggested that *L. (V.) guyanensis hsp70* copies are more heavily represented in the genotypes of the hybrids. The comparative analysis of the relative intensity between the 180 bp band from *L. (V.) guyanensis* and the 120 bp band from *L. (V.) shawi* showed that the heterozygous isolates presented a *L. (V.) guyanensis* band that was approximately 6x more intense. The 5 and 6 isolates presented three homozygous isoenzyme profiles, two of which were similar to that of *L. (V.) guyanensis.* The 3 isolate presented all the isoenzymes of a homozygous profile similar to that of *L*. (*V*.) *guyanensis*. These findings suggest that *L. (V.) guyanensis* accounts for a higher proportion of the phenotypic expression of the hybrids. However, other tests are necessary to confirm this hypothesis.

It is important to emphasize that different *L. (V.) shawi* and *L. (V.) guyanensis* strains from Pará State and the North region of Brazil included in our analysis (Table 1) presented the same RFLP and HRM profile, demonstrating that these targets are conserved among the strains used in this study, even between *L. (V.) shawi shawi* and *L. (V.) shawi santarensis* subpopulations. However, previous findings have suggested that the *L. (V.) shawi shawi* subpopulation is the probable the parental species (de Souza et al., 2016).

The present study confirmed by molecular methods, the existence of at least five strains of hybrid parasites *L*. (*V*.) *shawi* / *L*. (*V*.) *guyanensis* involved in cases of ATL in the Amazon region of Santarém Para, Brazil.

## Supplementary material

The following online material is available for this article:

Table S1 - Compiled data of *hsp70* and *mpi* PCR RFLP and multilocus enzyme electrophoresis (MLEE) from Jennings *et al.,* 2014.

Table S2 - NCBI sequences accession numbers.

Figure S1 - Illustrative gel showing *hsp70* and *mpi* PCR-RFLP (*Hae*III) products from cloned parasites fractionated on 3% agarose stained with GelRed.

Figure S2 - Alignment of 234 bp fragments used in *hsp70-*RFLP approach.

Figure S3 - Ratio between the intensity of the 180 bp band from *L. (V.) guyanensis* and the 120 bp band from *L. (V.) shawi* in heterozygous isolates normalized to a 1:1 ratio parental mixture.
