# Supplementary material for: Molecular tools confirm natural Leishmania (Viannia) guyanensis/L. (V.) shawi hybrids causing cutaneous leishmaniasis in the Amazon region of Brazil
Source: Genet Mol Biol. 2021 Apr 30;44(2):e20200123. doi: 10.1590/1678-4685-GMB-2020-0123 (PMC8108439; doi:10.1590/1678-4685-GMB-2020-0123)
Supplement: Table S1 - [file 1415-4757-GMB-44-2-e20200123-s1.pdf]

Genetics and Molecular Biology
https://doi.org/10.1590/1678-4685-GMB-2020-0123

Supplementary material to "Molecular tools confirmed the presence of natural *Leishmania (Viannia) guyanensis/L. (V.) shawi* hybrids causing cutaneous leishmaniasis in the Amazon region of Brazil"

Table S1 - Compiled data of *hsp70* and *mpi* PCR RFLP and multilocus enzyme electrophoresis (MLEE) from Jennings *et al*. 2014 (15)

Isolate	*L.(Viannia) sp.*	G6PD[1]	PGM[2]	ALAT[3]	*hsp70*[4]	*Mpi*[5]	Groups
	L.(V.) guyanensis	2	3	2	*L.(V.)g.*	*L.(V.)g.*	
	L.(V.) shawi shawi	1	4	2	*L.(V.)s.*	*L.(V.)s.*	
1	M15983	2	4	2	*L.(V.)s.*	*L.(V.)g.*	1
2	M15984	2	3	2	*L.(V.)s.*	hybrid	2
3	M15987	2	3	2	hybrid	hybrid	3
4	M15988	2	3	2	*L.(V.)s.*	*L.(V.)s.*	4
5	M19672	2	4	2	hybrid	hybrid	5
6	M19676	2	4	2	hybrid	hybrid	5
7	M19697	2	4	2	*L.(V.)s.*	*L.(V.) s.*	6

[1,2 e 3]: MLEE profiles of *L.* (*V.*) *guyanensis* and *L.* (*V.*) *shawi* species: IEC Zymodemes: Zymodemes Instituto Evandro Chagas profiles: G6PD[1]: Glucose-6-Phosphate Dehydrogenase; PGM[2]: Phosphoglycerate mutase; ALAT[3]: alanine aminotransferase; [4]*hsp70*: *hsp70*PCR-RFLP (*Hae*III) profile. *mpi*[5]*: mpi* PCR-RFLP (*ClaI*) profile.
