# Supplementary material for: Molecular tools confirm natural Leishmania (Viannia) guyanensis/L. (V.) shawi hybrids causing cutaneous leishmaniasis in the Amazon region of Brazil
Source: Genet Mol Biol. 2021 Apr 30;44(2):e20200123. doi: 10.1590/1678-4685-GMB-2020-0123 (PMC8108439; doi:10.1590/1678-4685-GMB-2020-0123)
Supplement: Table S2 - [file 1415-4757-GMB-44-2-e20200123-s2.pdf]

Genetics and Molecular Biology
https://doi.org/10.1590/1678-4685-GMB-2020-0123

Supplementary material to "Molecular tools confirmed the presence of natural *Leishmania (Viannia) guyanensis/L. (V.) shawi* hybrids causing cutaneous leishmaniasis in the Amazon region of Brazil"

Table S2 - NCBI sequences accession numbers

Leishmania sp.	Strain	NCBI Acession number
L.(V.) guyanensis M4147	MHOM/BR/1775/M4147	*MT337389*
L.(V.) guyanensis M19869	MHOM/BR/2001/M19869	*MT337390*
L.(V.) shawi M8408	MCEB/BR/1984/M8408	*MT337391*
L.(V.) shawi M19664	MHOM/BR/2001/M19664	*MT337392*
L.(V.) guyanensis/ L.(V.) shawi M15984	MHOM/BR/1996/M15984	*MT337393*
*L.(V.) guyanensis/ L.(V.) shawi M*15987	MHOM/BR/1996/M15987	*MT337394 / MT337395*
*L.(V.) guyanensis/ L.(V.) shawi M*15988	MHOM/BR/1996/M15988	*MT337396*
*L.(V.) guyanensis/ L.(V.) shawi M*19672	MHOM/BR/1996/M19672	*MT337397 / MT337398*
*L.(V.) guyanensis/ L.(V.) shawi M*19676	MHOM/BR/1996/M19676	*MT337399/ MT337400*
