# Supplementary material for: Molecular tools confirm natural Leishmania (Viannia) guyanensis/L. (V.) shawi hybrids causing cutaneous leishmaniasis in the Amazon region of Brazil
Source: Genet Mol Biol. 2021 Apr 30;44(2):e20200123. doi: 10.1590/1678-4685-GMB-2020-0123 (PMC8108439; doi:10.1590/1678-4685-GMB-2020-0123)
Supplement: Figure S1 - [file 1415-4757-GMB-44-2-e20200123-s3.pdf]

Genetics and Molecular Biology
https://doi.org/10.1590/1678-4685-GMB-2020-0123

Supplementary material to "Molecular tools confirmed the presence of natural *Leishmania (Viannia) guyanensis/L. (V.) shawi* hybrids causing cutaneous leishmaniasis in the Amazon region of Brazil"

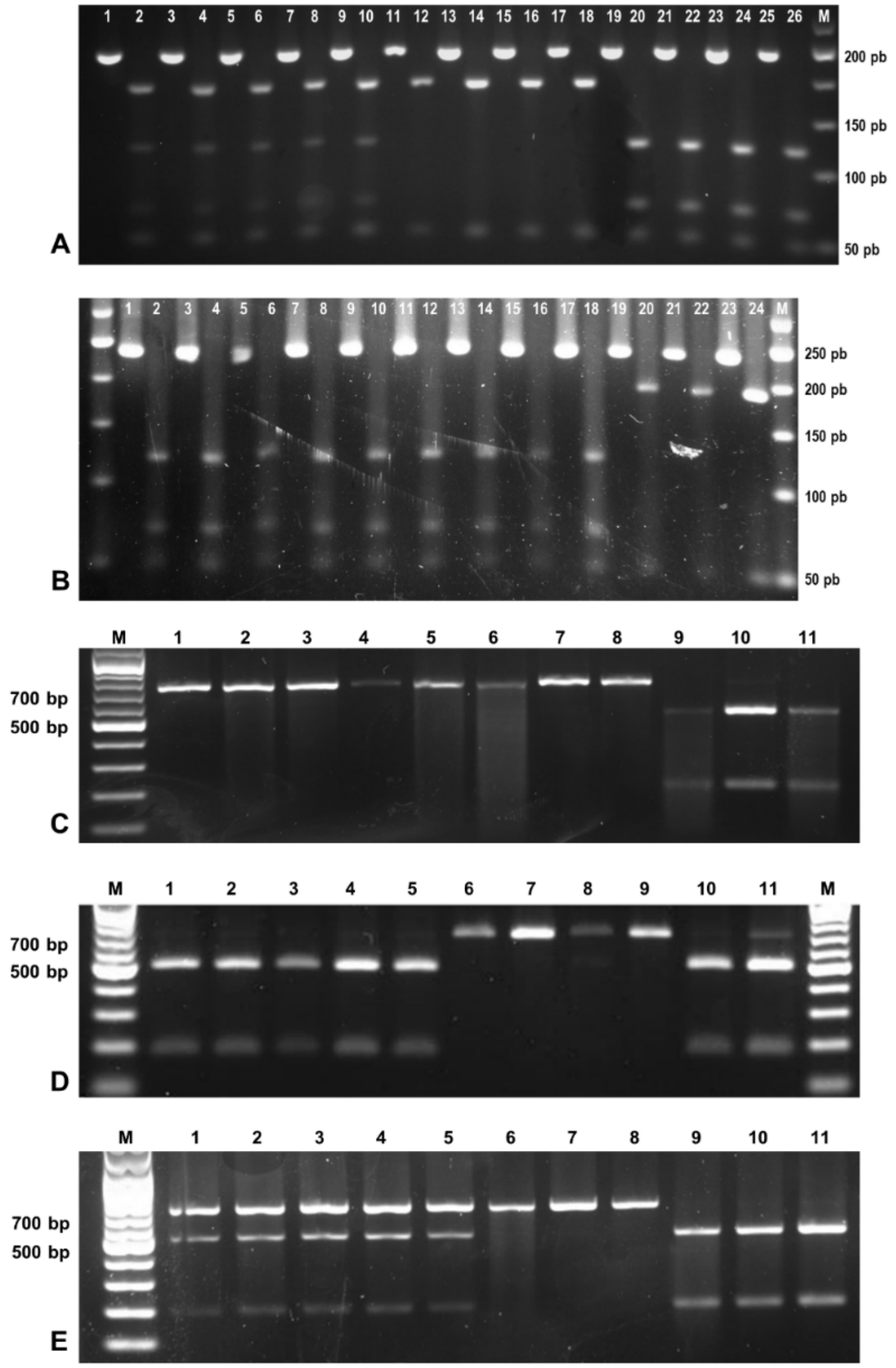

Figure S1- Illustrative gel showing *hsp70* and *mpi* PCR-RFLP (*Hae*III) products from cloned parasites fractionated on 3% agarose stained with GelRed

A: *hsp*70 PCR-RFLP heterozygous pattern (Isolate 5 /M19672). Odd lanes contain undigested PCR products, and even lanes contain PCR products digested with *Hae*III. Lanes 1 to 10 contain five clones isolated from M19672; Lanes 11 and 12: *L.* (*V.*) *guyanensis* (MHOM/BR/1990/M13245); Lanes 13 and 14: *L.* (*V.*) *guyanensis* (MHOM/BR/1975/M4147); Lanes 15 and 16 *L.* (*V.*) *guyanensis* (MHOM/BR/1997/M16174); Lanes 17 and 18: *L. (V.) guyanensis* (MHOM/BR/2001/M19869); Lanes 19 and 20: *L.* (*V.*) *shawi santarensis;* (MHOM/BR/1996/M15985); Lanes 21 and 22: *L. (V.) shawi shawi* (MCEB/BR/1984/**M8408); Lanes** 23 and 24: *L.* (*V.*) *shawi santarensis* (MHOM/BR/1996/M15982); Lanes 25 and 26: *L.* (*V.*) *shawi shawi* (MHOM/BR/2001/M19664); and M: 50 bp molecular weight marker (Fermentas SM 0373). **B:** *hsp70* PCR-RFLP homozygous pattern (Isolate 7/M19697). Lanes 1 to 10 contain five clones isolated from M19697; Lanes 11 and 12: *L. (V.) shawi shawi* (MHOM/BR/2001/M19664); Lanes 13 and 14: *L.* (*V.*) *shawi santarensis* (MHOM/BR/1996/M15982); Lanes 15 and 16: *L. (V.) shaw shawi* (MCEB/BR/1984/**M8408);** Lanes 17 and 18: *L. (V.) shawi santarensis* (MHOM/BR/1996/M15985); Lanes 19 and 20: *L. (V.) guyanensis* (MHOM/BR/1990/M13245); Lanes 21 and 22: *L.* (*V.*) *guyanensis* (MOM/BR/2001/M19869); Lanes 23 and 24: *L. (V.) guyanensis* (MHOM/BR/1975/M4147); **M**: 50 bp molecular weight marker (Fermentas SM 0373). **C:** *mpi* PCR-RFLP *L.* (*V.*) *shawi* homozygous pattern (Isolate 7/M19697/). M: 100 bp molecular weight marker (Fermentas SM 0331); Lanes 1 to 5: clones isolated from M19697**;** Lane 6: *L.* (*V.*) *shawi shawi* (MHOM/BR/2001/M19664); Lane 7: *L. (V.) shawi shawi* (MCEB/BR/1984/**M8408); Lane 8:** *L.* (*V.*) *shawi santarensis* (MHOM/BR/1996/M15982); Lane 9: *L.* (*V.*) *guyanensis* (MHOM/BR/1990/M13245), Lane 10: *L.* (*V.*) *guyanensis* (MHOM/BR/1975/M4147); Lane 11: *L.* (*V.*) *guyanensis* (MHOM/BR/1997/M16174). **D**: *mpi* PCR-RFLP *L.* (*V.*) *guyanensis* homozygous pattern (Isolate 1 /M15983). Lanes 1 to 5: clones isolated from M15983**;** Lane 6: *L.* (*V.*) *shawi shawi* (MHOM/BR/1996/M15982); Lane 7: *L. (V.) shawi shawi* (MCEB/BR/1984/**M8408); Lane 8:** *L.* (*V.*) *shawi santarensis* (MHOM/BR/2001/M19664); Lane 9: *L.* (*V.*) *shawi santarensis;* (MHOM/BR/1996/M15985); Lane 10: *L.* (*V.*) *guyanensis* (MHOM/BR/1975/M4147); Lane 11: *L.* (*V.*) *guyanensis* (MHOM/BR/1997/M16174) and M: 100 bp molecular weight marker (Fermentas SM 0331). **E:** *mpi* PCR-RFLP heterozygous pattern (Isolate 2/M15984). Lanes 1 to 5: clones isolate from M15984 that showed a heterozygous pattern. Lane 6: *L.* (*V.*) *shawi shawi* (MHOM/BR/2001/M19664); Lane 7: *L. (V.) shawi shawi* (MCEB/BR/1984/**M8408); Lane 8:** *L.* (*V.*) *shawi santarensis* (MHOM/BR/1996/M15982); Lane 9: *L.* (*V.*) *guyanensis* (MHOM/BR/1990/M13245), Lane 10: *L.* (*V.*) *guyanensis* (MHOM/BR/1975/M4147); Lane 11: *L.* (*V.*) *guyanensis* (MHOM/BR/1997/M16174) and **M:**100 bp molecular weight marker (Fermentas SM 0331).
