# Supplementary material for: Molecular tools confirm natural Leishmania (Viannia) guyanensis/L. (V.) shawi hybrids causing cutaneous leishmaniasis in the Amazon region of Brazil
Source: Genet Mol Biol. 2021 Apr 30;44(2):e20200123. doi: 10.1590/1678-4685-GMB-2020-0123 (PMC8108439; doi:10.1590/1678-4685-GMB-2020-0123)
Supplement: Figure S2 - [file 1415-4757-GMB-44-2-e20200123-s4.pdf]

Genetics and Molecular Biology
https://doi.org/10.1590/1678-4685-GMB-2020-0123

Supplementary material to "Molecular tools confirmed the presence of natural *Leishmania (Viannia) guyanensis/L. (V.) shawi* hybrids causing cutaneous leishmaniasis in the Amazon region of Brazil"

```
                          10        20        30        40        50        60        70
                  ....|....|....|....|....|....|....|....|....|....|....|....|....|....|
L.(V.)g M4147     GGACGAGATCGAGCGCATGGTGAACGATGCGTCGAAGTACGAGCAGGCCGACAAGATGCAGCGCGAGCGC
L.(V.)g.M19869    GGACGAGATCGAGCGCATGGTGAACGATGCGTCGAAGTACGAGCAGGCCGACAAGATGCAGCGCGAGCGC
L.(V.)s.M8408     GGACGAGATCGAGCGCATGGTGAACGATGCGTCGAAGTACGAGCAGGCCGACAAGATGCAGCGCGAGCGC
L.(V.)s.M19664    GGACGAGATCGAGCGCATGGTGAACGATGCGTCGAAGTACGAGCAGGCCGACAAGATGCAGCGCGAGCGC
M15984 sha        GGACGAGATCGAGCGCATGGTGAACGATGCGTCGAAGTACGAGCAGGCCGACAAGATGCAGCGCGAGCGC
M15987 sha        GGACGAGATCGAGCGCATGGTGAACGATGCGTCGAAGTACGAGCAGGCCGACAAGATGCAGCGCGAGCGC
M15987 guy        GGACGAGATCGAGCGCATGGTGAACGATGCGTCGAAGTACGAGCAGGCCGACAAGATGCAGCGCGAGCGC
M15988 sha        GGACGAGATCGAGCGCATGGTGAACGATGCGTCGAAGTACGAGCAGGCCGACAAGATGCAGCGCGAGCGC
M19672 guy        GGACGAGATCGAGCGCATGGTGAACGATGCGTCGAAGTACGAGCAGGCCGACAAGATGCAGCGCGAGCGC
M19672 sha        GGACGAGATCGAGCGCATGGTGAACGATGCGTCGAAGTACGAGCAGGCCGACAAGATGCAGCGCGAGCGC
M19676 guy        GGACGAGATCGAGCGCATGGTGAACGATGCGTCGAAGTACGAGCAGGCCGACAAGATGCAGCGCGAGCGC
M19676 sha        GGACGAGATCGAGCGCATGGTGAACGATGCGTCGAAGTACGAGCAGGCCGACAAGATGCAGCGCGAGCGC

                          80        90       100       110       120       130       140
                  ....|....|....|....|....|....|....|....|....|....|....|....|....|....|
L.(V.)g M4147     GTGGAGGCGAAGAACGGTCTGGAGAACTACGCGTACTCGATGAAGAACACGATCGCCGACACGAACGTGT
L.(V.)g.M19869    GTGGAGGCGAAGAACGGTCTGGAGAACTACGCGTACTCGATGAAGAACACGATCGCCGACACGAACGTGT
L.(V.)s.M8408     GTGGAGGCGAAGAACGGTCTGGAGAACTACGCGTACTCGATGAAGAACACGATCGCCGACACGAACGTGT
L.(V.)s.M19664    GTGGAGGCGAAGAACGGTCTGGAGAACTACGCGTACTCGATGAAGAACACGATCGCCGACACGAACGTGT
M15984 sha        GTGGAGGCGAAGAACGGTCTGGAGAACTACGCGTACTCGATGAAGAACACGATCGCCGACACGAACGTGT
M15987 sha        GTGGAGGCGAAGAACGGTCTGGAGAACTACGCGTACTCGATGAAGAACACGATCGCCGACACGAACGTGT
M15987 guy        GTGGAGGCGAAGAACGGTCTGGAGAACTACGCGTACTCGATGAAGAACACGATCGCCGACACGAACGTGT
M15988 sha        GTGGAGGCGAAGAACGGTCTGGAGAACTACGCGTACTCGATGAAGAACACGATCGCCGACACGAACGTGT
M19672 guy        GTGGAGGCGAAGAACGGTCTGGAGAACTACGCGTACTCGATGAAGAACACGATCGCCGACACGAACGTGT
M19672 sha        GTGGAGGCGAAGAACGGTCTGGAGAACTACGCGTACTCGATGAAGAACACGATCGCCGACACGAACGTGT
M19676 guy        GTGGAGGCGAAGAACGGTCTGGAGAACTACGCGTACTCGATGAAGAACACGATCGCCGACACGAACGTGT
M19676 sha        GTGGAGGCGAAGAACGGTCTGGAGAACTACGCGTACTCGATGAAGAACACGATCGCCGACACGAACGTGT

                         150       160       170       180       190       200       210
                  ....|....|....|....|....|....|....|....|....|....|....|....|....|....|
L.(V.)g M4147     CCGGCAAGCTGGAGGAGAGCGACAGGACCGCGCTGAACTCGGCGATCGACACGGCGCTGGAGTGGCTGAA
L.(V.)g.M19869    CCGGCAAGCTGGAGGAGAGCGACAGGACCGCGCTGAACTCGGCGATCGACACGGCGCTGGAGTGGCTGAA
L.(V.)s.M8408     CCGGCAAGCTGGAGGAGAGCGACAGGGCCGCGCTGAACTCGGCGATCGACGCGGCGCTGGAGTGGCTGAA
L.(V.)s.M19664    CCGGCAAGCTGGAGGAGAGCGACAGGGCCGCGCTGAACTCGGCGATCGACGCGGCGCTGGAGTGGCTGAA
M15984 sha        CCGGCAAGCTGGAGGAGAGCGACAGGGCCGCGCTGAACTCGGCGATCGACGCGGCGCTGGAGTGGCTGAA
M15987 sha        CCGGCAAGCTGGAGGAGAGCGACAGGGCCGCGCTGAACTCGGCGATCGACGCGGCGCTGGAGTGGCTGAA
M15987 guy        CCGGCAAGCTGGAGGAGAGCGACAGGACCGCGCTGAACTCGGCGATCGACACGGCGCTGGAGTGGCTGAA
M15988 sha        CCGGCAAGCTGGAGGAGAGCGACAGGGCCGCGCTGAACTCGGCGATCGACGCGGCGCTGGAGTGGCTGAA
M19672 guy        CCGGCAAGCTGGAGGAGAGCGACAGGACCGCGCTGAACTCGGCGATCGACACGGCGCTGGAGTGGCTGAA
M19672 sha        CCGGCAAGCTGGAGGAGAGCGACAGGGCCGCGCTGAACTCGGCGATCGACGCGGCGCTGGAGTGGCTGAA
M19676 guy        CCGGCAAGCTGGAGGAGAGCGACAGGACCGCGCTGAACTCGGCGATCGACACGGCGCTGGAGTGGCTGAA
M19676 sha        CCGGCAAGCTGGAGGAGAGCGACAGGGCCGCGCTGAACTCGGCGATCGACGCGGCGCTGGAGTGGCTGAA

                         220       230
                  ....|....|....|....|....
L.(V.)g M4147     CAGCAACCAGGAGGCGTCGAAGGA
L.(V.)g.M19869    CAGCAACCAGGAGGCGTCGAAGGA
L.(V.)s.M8408     CAGCAACCAGGAGGCGTCGAAGGA
L.(V.)s.M19664    CAGCAACCAGGAGGCGTCGAAGGA
M15984 sha        CAGCAACCAGGAGGCGTCGAAGGA
M15987 sha        CAGCAACCAGGAGGCGTCGAAGGA
M15987 guy        CAGCAACCAGGAGGCGTCGAAGGA
M15988 sha        CAGCAACCAGGAGGCGTCGAAGGA
M19672 guy        CAGCAACCAGGAGGCGTCGAAGGA
M19672 sha        CAGCAACCAGGAGGCGTCGAAGGA
M19676 guy        CAGCAACCAGGAGGCGTCGAAGGA
M19676 sha        CAGCAACCAGGAGGCGTCGAAGGA
```

Figure S2 - Alignment of 234 bp fragments used in *hsp70*-RFLP approach. The arrows represent GGCC *Hae*III restriction sites that distinguish *L.* (*V.*) *guyanensis* (M4147 and M19869) from *L.* (*V.*) *shawi,* (M8408 and M19664) according Graça *et al.*, 2012. Top numbers correspond to the nucleotide positions in the *hsp70* product sequence. The sequences were deposited in Genbank with the accession numbers MT337389 to MT337400.
