# Supplementary material for: Molecular tools confirm natural Leishmania (Viannia) guyanensis/L. (V.) shawi hybrids causing cutaneous leishmaniasis in the Amazon region of Brazil
Source: Genet Mol Biol. 2021 Apr 30;44(2):e20200123. doi: 10.1590/1678-4685-GMB-2020-0123 (PMC8108439; doi:10.1590/1678-4685-GMB-2020-0123)
Supplement: Figure S3 - [file 1415-4757-GMB-44-2-e20200123-s5.pdf]

Genetics and Molecular Biology
https://doi.org/10.1590/1678-4685-GMB-2020-0123

Supplementary material to "Molecular tools confirmed the presence of natural *Leishmania (Viannia) guyanensis/L. (V.) shawi* hybrids causing cutaneous leishmaniasis in the Amazon region of Brazil"

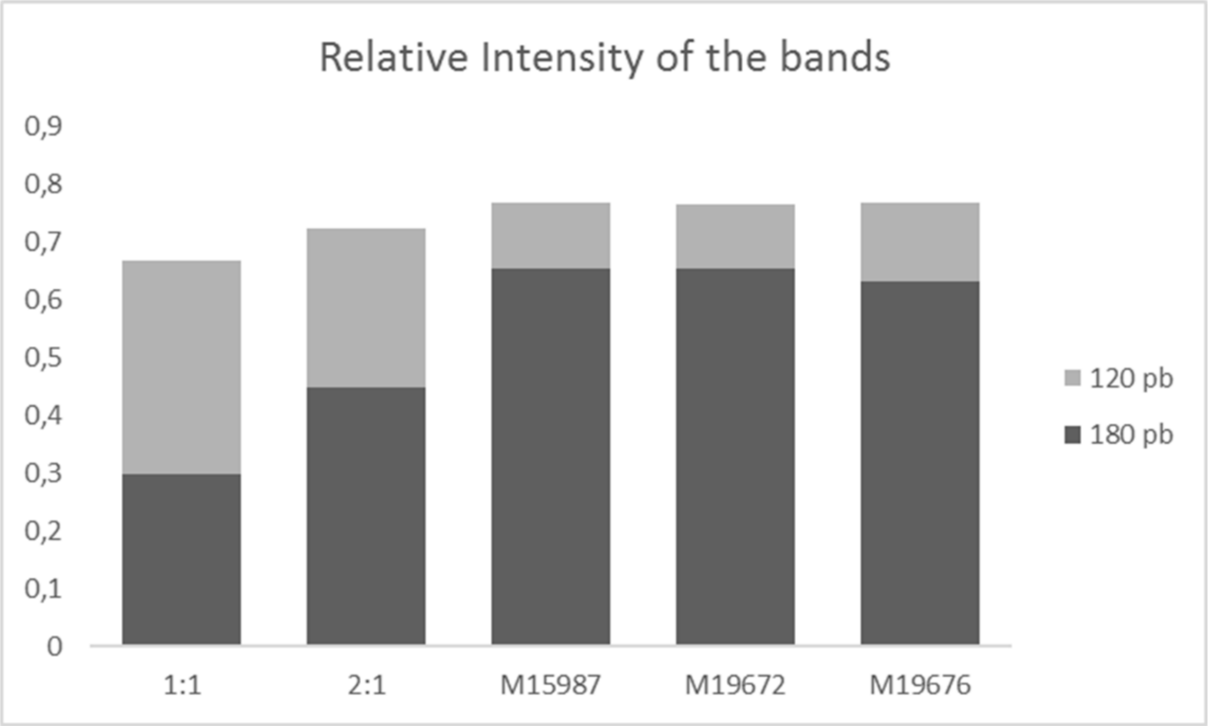

Figure S3 - Ratio between the intensity of the 180 bp band from *L. (V.) guyanensis* and the 120 bp band from *L. (V.) shawi* in heterozygous isolates normalized to a 1:1 ratio parental mixture.
